# Supplementary material for: The lncRNA SENCR knockdown alleviates vascular calcification via miR-4731-5p by suppressing endoplasmic reticulum stress
Source: PLoS One. 2025 May 12;20(5):e0323058. doi: 10.1371/journal.pone.0323058 (PMC12068569; doi:10.1371/journal.pone.0323058)
Supplement: Attachment — Submitted filename: Responses to Reviewers comments.doc [file pone.0323058.s002.doc]

**Responses to Reviewers' comments:**

Major points
1) The data presented are limited to an in vitro model of VC. To confirm the relevance of the SENRC/miR-4731-5p axis and propose it as a possible target for pharmacological intervention, the authors should verify the modulation of the two transcripts (SENRC/miR-4731-5p) in preclinical or clinical models (they could also analyse published data sets).

**Answer：Thank you for your suggestions.** Previous studies have demonstrated that overexpression of SENRC attenuated the proliferation, migration and phenotypic switching of vascular smooth muscle cells in AD patients and f atherosclerosis (AS). Furthermore, SENRC alleviated hypoxia/reoxygenation-induced cardiomyocyte apoptosis and inflammatory response in acute myocardial infarction. Especially, SENRC has been reported that its regulation in the malignant phenotype of AML cells by targeting miR-4731-5p. However, whether SENRC/miR-4731-5p involved in regulating osteogenic differentiation of VSMCs and VC remained unclear.

**References:**

1.[Yi Song](https://pubmed.ncbi.nlm.nih.gov/?term=Song+Y&cauthor_id=35351345), [Tao Wang](https://pubmed.ncbi.nlm.nih.gov/?term=Wang+T&cauthor_id=35351345), [Chunjie Mu](https://pubmed.ncbi.nlm.nih.gov/?term=Mu+C&cauthor_id=35351345), [Wenting Gui](https://pubmed.ncbi.nlm.nih.gov/?term=Gui+W&cauthor_id=35351345), [Yao Deng](https://pubmed.ncbi.nlm.nih.gov/?term=Deng+Y&cauthor_id=35351345), [Runwei Ma](https://pubmed.ncbi.nlm.nih.gov/?term=Ma+R&cauthor_id=35351345).LncRNA SENCR overexpression attenuated the proliferation, migration and phenotypic switching of vascular smooth muscle cells in aortic dissection via the miR-206/myocardin axis.Nutr Metab Cardiovasc Dis. 2022 Jun;32(6):1560-1570.

2.M. Chen, Y. Guo, Z. Sun, et al. Long non-coding RNA SENCR alleviates hypoxia/reoxygenation-induced cardiomyocyte apoptosis and inflammatory response by sponging miR-1.Cardiovasc Diagn. Ther., vol. 11 (3) (2021), pp. 707-715.

3.F. Ye, J. Zhang, Q. Zhang, et al. Preliminary study on the mechanism of long noncoding RNA SENCR regulating the proliferation and migration of vascular smooth muscle cells. J. Cell Physiol., vol. 235 (12) (2020), pp. 9635-9643.

4.Q. Lyu, S. Xu, Y. Lyu, et al. SENCR stabilizes vascular endothelial cell adherens junctions through interaction with CKAP4.Proc. Natl. Acad. Sci., vol. 116 (2) (2019), pp. 546-555.

5.[Changhao Han](https://pubmed.ncbi.nlm.nih.gov/?term=Han+C&cauthor_id=37120908), [Yan Qi](https://pubmed.ncbi.nlm.nih.gov/?term=Qi+Y&cauthor_id=37120908), [Yuanting She](https://pubmed.ncbi.nlm.nih.gov/?term=She+Y&cauthor_id=37120908), et al,.Long noncoding RNA SENCR facilitates the progression of acute myeloid leukemia through the miR-4731-5p/IRF2 pathway.Pathol Res Pract. 2023 May:245:154483.

**2) ENGLISH. The whole text would benefit from an English style revision. Several sentences are unclear or ambiguous. Just a few examples, lines 45-49 or 63-66, 248-250, 251-253, 253-254.**

**Answer：Thank you for your suggestions.** We have checked and revised in the manuscript. Thank you for bettering the quality about our manuscript.

Lines 265-267 please reconsider the sentence because two opposite effects of β-GP were proposed “the study revealed two novel findings: (1) excess β-GP increased SENRC levels by activating ERS during the procalcific process, and (2) β-GP-mediated downregulation of SENRC promoted the ERS-dependent osteogenic differentiation of VSMCs by sponging miR-4731-5p”.
**Answer：Thank you for your suggestions.** We have checked and revised in the manuscript. Thank you for bettering the quality about our manuscript.

Lines 240-242 please reconsider the sentence. The data description refers to fig 5C and D but not to fig 5B

**Answer：Thank you for your suggestions.** We have checked the sentence and added the data descriptions about the fig 5B, fig 5C and fig 5D in the manuscript. Thank you for bettering the quality about our manuscript.

3) DISCUSSION. Reconsider the discussion. There is often repetition in the text. For example, lines 271-274/276/277

The first part of the discussion is almost an introduction

The Limitation and conclusion section is unclear, please reformulate

**Answer：Thank you for your suggestions.** We have checked the possible duplicate parts and deleted those in order to integrity in the manuscript. Furthermore, we have reformulated in these parts. Thank you for bettering the quality about our manuscript.

4) REFERENCES. Some sentences are not supported by the relevant literature. Just a few examples:
Lines 79-81 “Given the close association between excess β-glycerol phosphate, SENRC, ERS, and VC,”

Other examples lines 263-265/267-269/280-281/282
Lines 306-307 Accumulating evidence suggests that SENRC binds to microR-4731-5p as a sponge to inhibit downstream effects via a β-GP-induced Runx2-dependent pathway. This sentence is very relevant to the paper, but there is no relative reference
Reference 30 does not refer to LncSENRC
Although the SENRC/miR-4731-5p axis has been implicated for the first time in VC, the functional interaction between SENRC and miR-4731-5p has already been predicted by https://doi.org/10.1016/j.prp.2023.154483. Not cited in the text.

**Answer：Thank you for your suggestions.** According to reviewers’ suggestions, we have checked and cited the proper and relative references in the revised manuscript.

**5**) DATA ANALYSIS AND PRESENTATION. Often the number of experimental replicates is 3/4, please report the individual values in the bars (at least for Figures 1-3).
Please indicate in each figure legend the type of statistical analysis performed (t-test, two-way ANOVA, etc.)
Representative blots do not always correspond to their quantification, especially those involving Gpr78.
Figure 2. There is an apparent inconsistency between mRNA and protein levels for Sm22. Sm22 increases as mRNA but decreases as protein. This data should be discussed.

**Answer：Thank you for your suggestions.**

In the section of DATA ANALYSIS AND PRESENTATION, the number of experimental replicates is 3 times and the values of the bar were ratios between the statistic values.

We have indicated the type of statistical analysis performed in the manuscript.

According to the blots about their quantification, especially those involving Gpr78, the values were ratios compared with control groups. We think there were no debates about the values. Furthermore we have provided all the bots for the experiments.

In figure 2, in the process of data statistics, we made some mistakes about the inconsistency between mRNA and protein levels, actually the changes in mRNA and protein levels are consistent. Sorry for disturbing you about confusion.

**6) PRESENTATION OF THE FIGURES. Figures 4 and 5 are very complex. To avoid confusion, please report the letter in the upper left corner of each panel. Also in figure 4, report the title for the 4 pictures in panel 4B**

Answer：Thank you for your suggestions. We have revised and represent the Figures in the revised manuscript including all the Figure 1,2,3,4,5. Thank you for bettering the quality about our manuscript.

Minor points
1) Please provide the complete image of the representative blots in the supplementary materials for referees. It would be preferable to present a couple of samples, rather than just one, as shown.

**Answer：Thank you for your suggestions.** We have provided the the complete image of the representative blots in the supplementary materials for referees

2) Abbreviations: Please define the acronyms on first mention within the text. Additionally, several acronyms remain unidentified (SENCR, TS1, etc.).

**Answer：Thank you for your suggestions.** We have checked in the manuscript.

**SENCR:**

Smooth muscle and endothelial cell-enriched migration/differentiation-associated [lncRNA](https://www.sciencedirect.com/topics/pharmacology-toxicology-and-pharmaceutical-science/long-untranslated-rna)

**TS1:**tumor stemness index (TSI); **ANCR:** lncRNA anti-differentiation noncoding RNA (ANCR)

VC is vascular calcification, not vascular calcium (abstract row 24).

**Answer：Thank you for your suggestions.** We have checked and revised in the manuscript.

3) Please ensure that the correct nomenclature of genes and proteins is used.

**Answer：Thank you for your suggestions.** We have checked the correct nomenclature of genes and proteins.

4) Row 200: This is probably fig 3A.

**Answer：Thank you for your suggestions.** We have revised the error in Row in the manuscript. The figure is actually figure 3A
